# Egg Yolk Protein Delays Recovery while Ovalbumin Is Useful in Recovery from Iron Deficiency Anemia

**DOI:** 10.3390/nu7064792

**Published:** 2015-06-15

**Authors:** Yukiko Kobayashi, Etsuko Wakasugi, Risa Yasui, Masashi Kuwahata, Yasuhiro Kido

**Affiliations:** Laboratory of Nutrition Science, Graduate School of Life and Environmental Sciences, Kyoto Prefectural University, Shimogamo, Sakyo, Kyoto 606-8522, Japan; E-Mails: hgjwj757@yahoo.co.jp (E.W.); kbysykk@gmail.com (R.Y.); kuwahata@kpu.ac.jp (M.K.); kido@kpu.ac.jp (Y.K.)

**Keywords:** dietary protein, iron deficiency anemia, egg-yolk protein, ovalbumin

## Abstract

Protein is a main nutrient involved in overall iron metabolism *in vivo*. In order to assess the prevention of iron deficiency anemia (IDA) by diet, it is necessary to confirm the influence of dietary protein, which coexists with iron, on iron bioavailability. We investigated the usefulness of the egg structural protein in recovery from IDA. Thirty-one female Sprague-Dawley rats were divided into a control group (*n* = 6) fed a casein diet (4.0 mg Fe/100 g) for 42 days and an IDA model group (*n* = 25) created by feeding a low-iron casein diet (LI, 0.4 mg Fe/100 g) for 21 days and these IDA rats were fed normal iron diet with different proteins from eggs for another 21 days. The IDA rats were further divided into four subgroups depending on the proteins fed during the last 21 days, which were those with an egg white diet (LI-W, 4.0 mg Fe/100 g, *n* = 6), those with an ovalbumin diet (LI-A, 4.0 mg Fe/100 g, *n* = 7), those with an egg yolk-supplemented diet (LI-Y, 4.0 mg Fe/100 g, *n* = 6), and the rest with a casein diet (LI-C, 4.0 mg Fe/100 g, *n* = 6). In the LI-Y group, recovery of the hematocrit, hemoglobin, transferrin saturation level and the hepatic iron content were delayed compared to the other groups (*p* < 0.01, 0.01, 0.01, and 0.05, respectively), resulting in no recovery from IDA at the end of the experimental period. There were no significant differences in blood parameters in the LI-W and LI-A groups compared to the control group. The hepatic iron content of the LI-W and LI-A groups was higher than that of the LI-C group (*p* < 0.05). We found that egg white protein was useful for recovery from IDA and one of the efficacious components was ovalbumin, while egg yolk protein delayed recovery of IDA. This study demonstrates, therefore, that bioavailability of dietary iron varies depending on the source of dietary protein.

## 1. Introduction

Protein is one of the main nutrients involved in all aspects of *in vivo* iron metabolism including iron absorption, transport, hematopoiesis and storage [1,2]. Thus, adequate intake of not only iron, but also protein, is important in the maintenance of normal iron metabolism. Dietary protein differs in the composition of amino acids and the amino acid score by the food origin. Moreover, peptides, which are the digestive form of proteins, are thought to modulate biological functions, and have recently been shown to have various physiological functions. In assessing prevention of iron deficiency anemia (IDA) by diet, it is therefore necessary to confirm the influence and biological use of dietary protein, which coexists with iron, on iron absorption.

Of the three major nutrients (carbohydrate, protein, and lipid), protein has been reported to have the greatest influence on iron absorption [3,4,5,6]. For example, Cook *et al.* reported that soybean protein inhibited iron absorption more than egg white protein by a factor of about 5 [7]. Although the whole egg is a food that contains a large amount of iron [8], it is often cited as a food that inhibits iron absorption [9,10,11]. The majority of iron in eggs is found in the yolk. Although it has been reported that the absorptivity of iron from egg origin is low [12,13], iron absorption was shown to increase and contribute to a delay in the decrease in hemoglobin concentration in an iron-deficient state where iron demands were high [14]. The mineral absorption promoting effect of casein phosphopeptide (CPP) of milk origin is well known from the past researches [15,16,17]. Although a number of studies have examined the influence of dietary protein on iron absorption, there are few reports on the contribution of dietary protein in the iron deficient state that is accompanied by abnormal iron metabolism [3,4,5,6,7,8,9,10,11,12,13,14,15,16,17].

We previously examined the effects of various dietary protein sources consumed simultaneously with dietary iron on recovery from IDA. Our previous research showed the possibility that egg white contributes to prompt recovery from IDA compared to soybeans. Furthermore, we showed that the protein form resulted in an increase in serum iron and maturation of red blood cells compared to the peptide form, because the protein form maintained its iron-reducing characteristics in the digestive tract, compared to the peptide form [18]. In the present study, therefore, we investigated the usefulness of the egg structural protein for recovery from IDA.

## 2. Experimental Section

### 2.1. Animal Experimental Protocol

This experimental study was approved by the ethics committee of Kyoto Prefectural University, and performed in accordance with the Guidelines for Animal Experimentation at Kyoto Prefectural University. Thirty-one 4-week-old female Sprague-Dawley rats were used in this study (Japan SLC, Inc., Hamamatsu, Japan). The rats were individually housed in stainless steel cages at a controlled temperature of 22–24 °C, a relative humidity of 40%–60%, and a light cycle of 12 h with free access to distilled water (the iron content of the distilled water was previously measured). Body weight and food intake were recorded at the same time everyday.

The 31 rats were divided into two groups on the basis of body weight. The first group (C group, *n* = 6, weighing 106–112 g) was fed a control diet for 42 days. The second group (base LI group, *n* = 25, weighing 94–121 g) was fed a low-iron diet for 21 days to induce IDA. IDA rats were then divided into four subgroups based on weight and hemoglobin concentration such that the mean values of these parameters for each subgroup were the same. Each subgroup was fed either an egg white diet (LI-W group, *n* = 6, weighing 155–195 g), an ovalbumin diet (LI-A group, *n* = 7, weighing 153–177 g), an egg-yolk supplemented diet (LI-Y group, *n* = 6, weighing 158–184 g), or the control diet (LI-C group, *n* = 6, weighing 163–182 g) for another 21 days. The compositions of the diets used in the experiments are shown in Table 1. All diets were prepared according to the AIN-76 formulation with one modification (the addition of choline chloride). The low-iron diet contained 0.4 mg Fe/100 g without any ferrous citrate in the mineral mixture. The amount of protein and lipids within all experimental diets was adjusted to be equal to that of the control diet. During the pair- feeding period, the LI-W, LI-A and LI-Y groups were provided with the same amount of diet that was freely provided to the LI-C group, on the following day.

**Table 1:** Composition of experimental diet.

	Control Diet	Low Iron Diet	Egg White Diet	Ovalbumin Diet	Egg York Diet
	(g/kg)				
Casein ^a^	200	200	-	-	171
Egg White powder ^b^	-	-	214	-	-
Ovalbumin ^c^	-	-	-	211	-
Egg York powder ^d^	-	-	-	-	81
α-starch	457	457	447	448	455
Sucrose	228	228	224	225	228
Mixed oil ^e^	50	50	50	50	
Vitamin mixture ^f^			10		
Mineral mixture ^g^			35		
Cellose			20		
	(mg/kg)				
Iron (III) Citrate	204	-	205	196	163
Iron content	39.6	4.4	40.0	39.9	40.0

^a^ 13.73 gN/100 g. ^b^ 12.97 gN/100 g. ^c^ 13.10 gN/100 g. ^d^ 4.91 gN/100 g. ^e^ Rapeseed oil/soybean oil ratio = 7/3. ^f^ AIN-76 vitamin mixture (per g mixture): vitamin A, 400 IU; vitamin D_3_, 100 IU; vitamin E, 5 mg; vitamin K_3_, 0.005 mg; vitamin B_1_, 0.6 mg; vitamin B_2_, 0.6 mg; vitamin B_6_, 0.7 mg; vitamin B_12_, 0.001 mg; D-biotin, 0.02 mg; folic acid, 0.2 mg; calcium pantothenate, 1.6 mg; nicotinic acid, 3 mg; choline chloride, 200 mg; sucrose, 0.968 g. ^g^ AIN-76 mineral mixture (g/kg mixture): calcium phosphate dibasic, 500.0; sodium chloride, 74.0; potassium citrate, 220.0; potassium sulfate, 52.0; magnesium oxide, 24.0; manganese carbonate, 3.5; zinc carbonate, 1.6; cupric carbonate, 0.3; potassium iodate, 0.01; sodium selenite, 0.0066; chromium potassium sulfate, 0.55; sucrose, 124.03.

Blood was drawn from the tail vein of all of the animals every 4 days during the experimental period. At the end of each study period, the rats were euthanized under ether anesthesia during the early phase of the light cycle in a non-fasted state, and blood samples drawn from the inferior vena cava were collected in tubes with heparin. Samples from the liver and small intestinal mucosa (upper side, 1/4th) were also collected.

### 2.2. Blood Constituent Analysis

The blood hemoglobin concentration was measured using Hemoglobin B test Wako (Wako Pure Chemical Industries, Osaka, Japan). The hematocrit level was measured after centrifugation of the blood at 12,000 rpm for 5 min at 4 °C. Red blood cell (RBC) counts were determined using a Thoma hemacytometer following a 1:200 dilution with Hayem’s solution in a pipette. Mean cell volume (MCV), mean corpuscular hemoglobin (MCH) and mean cell hemoglobin concentration (MCHC) were calculated as follows:

MCV (pg) = Hb (g/dL)/RBC (×10^6^/μL) × 10 (1)

MCH (fL) = Ht (%)/RBC (×106/μL) × 10 (2)

MCHC (%) = Hb (g/dL)/Ht (%) × 100 (3)

Serum iron and unsaturated iron binding capacity (UIBC) were measured using Detaminer Fe and UIBC (Kyowa Medix Co., Ltd., Tokyo, Japan) with an automatic biochemical analyzer (CL-8000; Shimadzu Corp., Kyoto, Japan). Total iron binding capacity and serum transferrin saturation were calculated as follows:

Total iron binding capacity = serum iron + UIBC (4)

Serum transferrin saturation = serum iron/Total iron binding capacity × 100 (5)

### 2.3. Estimation of Gene Expression

Total RNA was isolated from the homogenized mucosa and liver samples using the Total RNA Isolation mini kit (Agilent Technologies, Inc., Santa Clara, CA, USA), and converted to cDNA using a reverse transcriptase enzyme ReverTra Ace (Toyobo Co., Ltd., Osaka, Japan) according to the manufacturer’s instructions. Each target DNA fragment was amplified using the respective TaqMan gene expression assay kits and a real-time polymerase chain reaction (PCR) system using cDNA as a template. Real-time PCR for gene expression analysis was performed using DNA Engine Opticon and Opticon Monitor software (Bio-Rad Laboratories, Inc., Hercules, CA, USA). TaqMan primer pairs/probes for gene analysis were obtained using a TaqMan Gene Expression Assay (Applied Biosystems, Inc., Carlsbad, CA, USA). Assay IDs were Rn00565927_m1, Rn00591187_m1 and Rn00667869_m1 for DMT1, Ferroportin and β-actin, respectively. Reactions were performed with 10 μL of Premix EX Taq (Takara Bio, Inc., Ohtsu, Japan), 1 μL of the primer pairs/probes sets and 3 μL of cDNA in a final volume of 20 μL. After heating the test sample at 96 °C for 10 s, 50 PCR cycles were performed as follows: 95 °C for 7 s, 60 °C for 30 s, and 72 °C for 20 s. The cycle thresholds of the genes of interest were compared with the housekeeping gene β-actin to determine relative changes in expression.

### 2.4. Iron Content of Hepatic Tissue

Liver samples were perfused with saline, and treated by the wet ash method using a microwave extraction system (Ethos; Milestone Srl., Sorisole, Italy). The ash was suspended in dilute hydrochloric acid solution after evaporation, and left to dry. Iron concentrations were measured by polarizing Zeeman-effect atomic absorption spectrometry (Z-6100; Hitachi, Ltd., Tokyo, Japan) after suitable dilution. We determined that the coefficient of variation was 0.04. Iron concentrations were expressed on a wet-weight basis.

### 2.5. Statistical Analysis

**Figure 1:**
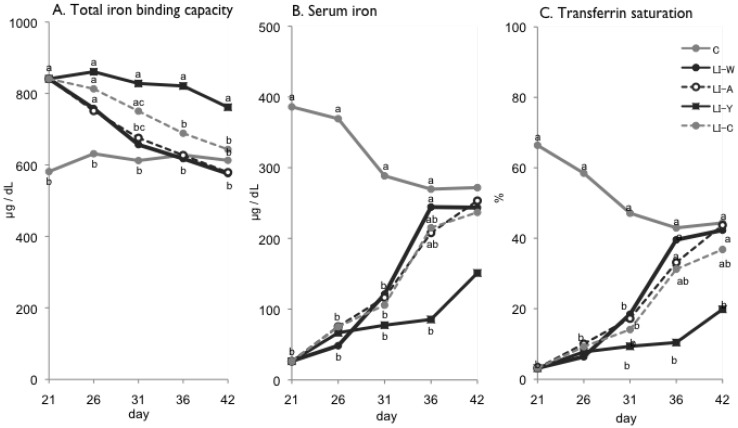
Total iron binding capacity (**A**); serum iron (**B**); transferrin saturation (**C**) on day 21, 26, 31, 36 and 42 after start of experimental period. Iron deficiency anemia rats, fed low iron diet for 21 days, divided four subgroups, was fed either an egg white diet (LI-W, *n* = 6), an ovalbumin diet (LI-A, *n* = 7), an egg york diet (LI-Y, *n* = 6) or a control diet (LI-C, *n* = 6) and normal rats fed the control diet (C, *n* = 6) for 42 days. Values were represented as mean. Values with an unlike letter were significant: *p* < 0.05.

**Figure 2:**
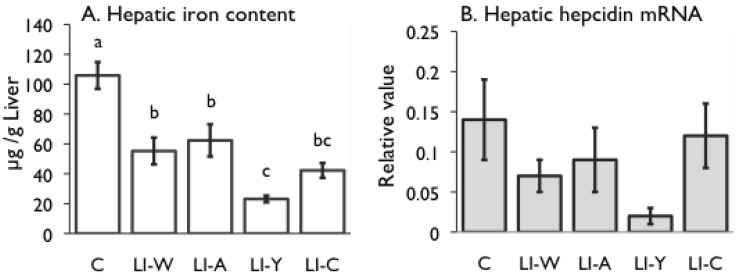

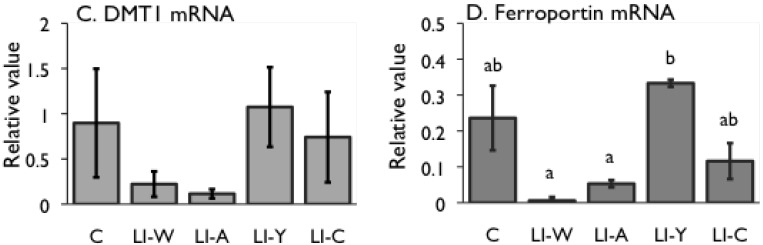
Iron content (**A**) and hepcidin mRNA expression (**B**) in the liver, and iron transporter DMT1 (**C**) and Ferroportin (**D**) mRNA expression in small intestines on day 42 after start of study. Iron deficiency anemia rats, fed low iron diet for 21 days, divided four subgroups, was fed either an egg white diet (LI-W, *n* = 6), an ovalbumin diet (LI-A, *n* = 7), an egg york diet (LI-Y, *n* = 6) or a control diet (LI-C, *n* = 6) and normal rats fed the control diet (C, *n* = 6) for 42 days. The cycle thresholds of the genes of interest were compared with the housekeeping gene β-actin to determine relative changes in expression. Values were represented as means ± SEM. Values with unlike letter were significant: *p* < 0.05.

Data were presented as means ± standard error (SEM). Before assessing the different variables, we carried out a Bartlett test to check the normal distribution of the variables. Data that fit the normal distribution were compared by 1–way analysis of variance (ANOVA) followed by the Tukey-Kramer test (Table 2, Figure 1 and Figure 2), or Student’s t test (Table 2). The level of significance was set at *p* < 0.05.

**Table 2:** Body weight gain, food intake and blood parameters on the day 21 and day 42 after the start of study.

**A. day 21**						
	Base LI	C	Student’s *t*-test *p* value			
Body weight gain (g/day)	3.0 ± 0.1	3.4 ± 0.2	0.138			
Food intake (g/day)	11.7 ± 0.1 ^a^	12.7 ± 0.4 ^b^	0.034			
Hematocrit level (%)	33.0 ± 0.7 ^a^	47.1 ± 1.2 ^b^	<0.001			
Hemogrobin concentration (g/dL)	9.7 ± 0.3 ^a^	15.4 ±0.7 ^b^	<0.001			
**B. day 42**						
	LI-W	LI-A	LI-Y	LI-C	C	ANOVA *p* value
Body weight gain (g/day)	1.4 ± 0.3	1.5 ± 0.1	1.3 ± 0.1	1.5 ± 0.2	1.7 ± 0.1	0.196
Food intake (g/day)	10.9 ± 0.0 ^a^	10.9 ± 0.0 ^a^	10.9 ± 0.0 ^a^	10.9 ± 0.0 ^a^	11.9 ± 0.5 ^b^	0.006
Hematocrit level (%)	49.9 ± 1.5 ^a^	49.3 ± 1.0 ^a^	44.4 ± 3.2 ^b^	48.5 ± 2.8 ^a^	50.7 ± 1.0 ^a^	0.002
Hemogrobin concentration (g/dL)	18.0 ± 0.6 ^a^	17.8 ± 0.6 ^a^	15.0 ± 0.6 ^b^	17.4 ± 1.0 ^a^	18.2 ± 0.5 ^a^	<0.001
Red blood cell counts (×10^6^/µL)	14.8 ± 1.1	13.0 ± 1.5	13.5 ± 1.3	14.1 ± 1.8	15.8 ± 2.2	0.072
Mean cell volume * (fL)	12.0 ± 0.4 ^ab^	13.8 ± 1.8 ^a^	11.2 ± 1.1 ^b^	12.4 ± 0.7 ^ab^	11.7 ± 2.0 ^ab^	0.013
Mean corpuscular hemoglobin ** (pg)	33.2 ± 1.0 ^a^	38.4 ± 4.0 ^b^	33.3 ± 4.5 ^a^	34.4 ± 2.0 ^a^	31.6 ± 4.7 ^a^	0.043
Mean cell hemogrobin concentration *** (%)	36.0 ± 1.6 ^ab^	39.0 ± 1.1 ^ab^	33.7 ± 1.4 ^a^	31.4 ± 0.3 ^ab^	36.0 ± 0.6 ^b^	0.022

Normal rats fed low iron diet (base LI, *n* = 25) for 21 days or control diet (C, *n* = 6) for 42 days. Base LI group divided four subgroups, were fed either an egg white diet (LI-W, *n* = 6), an ovalbumin diet (LI-A, *n* = 7), an egg york-supplemented diet (LI-Y, *n* = 6) or control diet (LI-C, *n* = 6) for another 21 days. Values are mean ± SEM. Values with an unlike letter were significant: *p* < 0.05. * Mean cell volume (pg) = Hb (g/dL)/RBC (×10^6^/µL) × 10, ** Mean corpuscular hemoglobin (fL) = Ht (%)/RBC (×10^6^/µL) × 10, *** Mean cell hemogrobin concentration (%) = Hb (g/dL)/Ht (%) × 100.

## 3. Results

### 3.1. Body Weight Gain, Food Intake and Blood Parameters

Body weight gain, food intake and blood parameters on day 21 and day 42 after the start of the study are shown in Table 2. There was no significant difference in body weight gain or food intake between the two base groups on day 21. The hematocrit level and hemoglobin concentration of the base LI group (LI-W, LI-A, LI-Y and LI-C group) were lower than those of the C group on day 21 (hematocrit: 33.0 ± 0.7 *vs.* 47.1 ± 1.2%; *p* < 0.001, hemoglobin: 9.7 ± 0.3 *vs.* 15.4 ± 0.7 g/dL; *p* < 0.001), confirming that the base LI group had developed IDA. On day 42 after the start of the study, the hematocrit level and hemoglobin concentration of the LI-Y group were significantly lower than that of the other four groups (Table 2, hematocrit: LI-Y 44.4 ± 3.2 *vs.* C 50.7 ± 1.0 and LI-C 48.5 ± 2.8%; *p* < 0.05, LI-Y *vs.* LI-A 49.3 ± 1.0 and LI-W 49.9 ± 1.5%; *p* < 0.01, hemoglobin: LI-Y 15.0 ± 0.6 *vs.* LI-W 18.0 ± 0.6, LI-A 17.8 ± 0.6, LI-C 17.4 ± 1.0 and C 18.2 ± 0.5 g/dL; *p* < 0.01 for all); however, no statistical difference was found among the LI-W, LI-A, LI-C, and C groups. The MCV of the LI-Y group was significantly lower than that of the LI-A group (*p* < 0.05) and the MCH of the LI-A group was higher than that of the other four groups (*p* < 0.05). There were no significant differences in body weight gain, food intake, RBC and MCHC among four groups of base LI.

### 3.2. The Changes in the Total Iron Binding Capacity, Serum Iron and Transferrin Saturation Level of the Groups

Figure 1 shows the changes in the total iron binding capacity, serum iron and transferrin saturation level of the groups on days 21, 26, 31, 36 and 42 after the study. On day 21, the base LI group (LI-W, LI-A, LI-Y and LI-C group) exhibited lower serum iron and transferrin saturation and higher total iron binding capacity compared to the C group (*p* < 0.01 for all). The total iron binding capacity, serum iron and transferrin saturation level of the base LI group fed the diets containing iron were gradually restored. The total iron binding capacity of the LI-Y group was statistically higher than that of the C group from day 21 to day 42 (*p* < 0.01 for all). From day 21 to day 31, the total iron binding capacity of the LI-C group was higher than that of the C group (*p* < 0.01 for all); however, there were no significant differences between the LI-W, LI-A and C groups on day 31. There were also no statistical differences among the LI-W, LI-A and LI-C groups after day 21, until day 42 (Figure 1A). The serum iron level of the LI-Y group was statistically lower than that of the C group from day 21 to day 36 (*p* < 0.01 for all). There were no significant differences among the LI-W, LI-A, LI-C and C groups on day 36 and no significant differences between LI-W, LI-A and LI-C after day 21 to the end of the experiment (Figure 1B). The transferring saturation level of LI-Y group was statistically lower than that of the C group from day 21 to day 42 (*p* < 0.01 for all). There were no significant differences among the LI-W, LI-A, LI-C and C groups on day 36 (Figure 1C).

### 3.3. The Hepatic Iron Content and the mRNA Expression Level of Liver and Mucos

The hepatic iron content of the base LI group (LI-W: 55.2 ± 9.0, LI-A: 62.3 ± 10.8, LI-Y: 23.2 ± 2.2 and LI-C: 42.2 ± 4.9 µg/g liver) was much lower than that of the C group (73.7 ± 20.3 µg/g liver) on day 42. The iron content of hepatic tissue of the LI-W and LI-A groups was dramatically increased compared with that of the LI-Y group (*p* < 0.05), and higher than that of the LI-C group but no significant differences were observed among the three groups (Figure 2A). A correlation between the mRNA expression level of hepatic hepcidin and hepatic iron content was observed in both groups (Figure 2B–D). The hepcidin mRNA expression level of the LI-Y group was lower compared with the other groups, but no significant difference was shown among five groups (Figure 2B). The DMT1 mRNA expression levels of the LI-W, LI-A and LI-C groups were lower than those of the C group. In contrast, the mRNA expression level of the LI-Y group was increased compared with the C group, but there were no statistical differences between the C and the other groups (Figure 2C). The pattern of expression levels of ferroportin mRNA was similar to the DMT1 mRNA expression levels (Figure 2D).

## 4. Discussion

In the present study, we examined the usefulness of egg constitutive proteins on recovery of IDA. Our results provided new finding that egg yolk protein delayed recovery of IDA while ovalbumin was useful in recovery of IDA, and that the bioavailability of dietary iron varies depending on dietary protein source.

Although the iron content of all diets was equivalent, the transitions of total iron binding capacity, transferring saturation level, and serum iron in the LI-Y group of IDA rats, which were fed the diet containing egg yolk, showed delayed recovery compared with the other groups, and resulted in no recovery at the end of the experimental period. Moreover, the blood properties and hepatic iron content of the LI-Y group were lower than those of the other groups on day 42. These data suggest that egg yolk resulted in delayed recovery from IDA. On the other hand, the mRNA expression of hepatic hepcidin, which regulates iron absorption in the gut [19,20], was decreased in the LI-Y group compared to the other diet groups, and it correlated with hepatic iron content. The mRNA expression of ferroportin and DMT1, which are transporters of iron absorption in the small intestine [21,22], were up-regulated in the LI-Y group compared to the C group, suggesting that in this group, iron storage was insufficient and iron absorption was promoted by homeostasis. Nevertheless, expression of the iron transporters was up-regulated. One reason for the lack of recovery from IDA in the Li-Y group may be that the absolute quantity of dietary iron transported in the small intestines was low. A previous study reported that intake of egg yolk protein decreased the apparent absorption of iron, calcium and magnesium compared with casein and soy protein in normal rats [23]. Most of the iron in egg yolk is combined with phosvitin of phosphate protein. Phosvitin is known to have a very high binding capacity for divalent metals, especially iron [24]. It was observed that the amount of insoluble iron in the small intestines of rats fed the diet containing phosvitin was higher than that in rats fed diets without phosvitin [25]. In the present study, feeding the diet containing egg yolk may have delayed recovery from IDA because the iron in the egg yolk iron and/or other dietary iron may have strongly combined with phosvitin, and formed an insoluble iron complex in the small intestine. Since phosvitin is a resistant protein [26], it is possible that the insoluble iron complex was excreted from the body without being used *in vivo*. These findings suggest, therefore, that iron from eggs is not readily used *in vivo*, and ingredients from egg yolk reduce the bioavailability of dietary iron. In cases of IDA, the choice of egg yolk as the source of protein and iron should be avoided.

Conversely, at the end of the experimental period, the blood parameters of the LI-W group, which was fed egg white, the LI-A group, which was fed ovalbumin, and the LI-C group, which was fed casein, were not significantly different from those of the control group, suggesting that IDA improved in these groups. The hepatic iron content of the LI-W, LI-A, and LI-C groups was significantly lower than the C group, however, and iron storage was not recovered in these groups despite receiving the standard amount of dietary iron given to IDA rats for 3 weeks. The total iron binding capacity of the LI-W and LI-A group was lower than that of the LI-C, but was not significantly different from the C group. In addition, the hepatic iron content of the LI-W and LI-A groups was higher than that of the LI-C group, but there were no significant differences among the three groups. These findings show that IDA of the LI-W and LI-A groups, which were fed egg white protein, was promptly recovered due to inhibition of transferrin production at an early stage compared with the LI-C group, which was fed casein protein. We conclude, therefore, that the egg white protein contributed to an improvement in the iron deficient state compared to casein protein. These results are in agreement with our previous reports [18]. If iron absorption in the LI-C group, which was fed casein protein, was promoted by CPP, this raises the possibility that egg white protein has the absorption promoting effect on iron that exceeds CPP. On the other hand, a previous study reported that the specific amino acid that accelerated iron absorption exists in animal protein, such as red meat [27]. These results suggest that the mechanism for promotion of recovery from IDA by egg white protein differs from the rise in iron absorption caused by red meat, since the existence of this amino acid in egg has not been reported even though eggs are from animal sources. Moreover, we assume that the component of egg white protein active in recovery of IDA was ovalbumin, because there were no significant differences in changes in blood parameters and in hepatic iron content between the LI-W and LI-A groups. Albumin possesses a large number of negative carboxylate sites (−CO_2_^−^) on the surface of the molecule, many clustered in groups of three or more [28]. These sites are suitable for binding iron (III) [29]. Indeed, albumin has been demonstrated to be a sufficiently powerful ligand for binding iron (III) even when transferrin is not fully saturated [30]. Therefore, the combination of dietary iron and albumin in the lumen may form a soluble complex that is advantageous for iron absorption.

Despite the fact that the amount of stored iron in the LI-W, LI-A, and LI-C groups was not restored, the ferroportin and DMT1 mRNA levels in the small intestine significantly decreased in these groups compared with the C group. Accordingly, iron absorption by the transporters was suppressed in the LI-W, LI-A, and LI-C groups, even though they were in an iron-deficient state. It is known that the expression of the iron transporter ferroportin is down-regulated by secretion of hepcidin [31]; however, there was no correlation between hepatic iron content and the level of hepatic hepcidin and ferroportin mRNA expression in this study. Thus, these findings raise the possibility of the presence of another mechanism for regulation of ferroportin expression in the absence of secretion of hepcidin.

One of the limitations of the present study is that we did not directly measure the rate of iron absorption as the trend of dietary iron in the intestine is one of clues to comprehend the usefulness of the dietary iron for the recovery of IDA. In order to elucidate the effects of egg protein on the promotion of iron absorption, further studies should be conducted to investigate iron balance. Moreover, our findings suggest that phosvitin, contained within egg yolk protein, likely reduces the bioavailability of dietary iron. Further research is required to investigate the role of egg yolk phosvitin in the recovery of IDA and its possible usefulness in iron removal therapy. Although the results obtained in this study have some implications for public health, it is necessary to keep in mind that the iron bioavailability varies with different cooking processes, especially of egg yolk.

## 5. Conclusions

We demonstrated that the origin of dietary protein influenced iron absorption and maturation of red blood cells without affecting iron transport and hematogenesis in recovery from IDA. In particular, we showed that egg white protein was useful for recovery from IDA, and one of the efficacious components was ovalbumin and egg yolk protein delayed recovery of IDA. This study demonstrates, therefore, that bioavailability of dietary iron varies depending on the source of dietary protein. Further analysis of the mechanism for IDA recovery by ovalbumin may lead to the development of a new diet therapy for IDA such as thalassaemias, that does not include iron loading. Conversely, egg yolk protein may be useful in iron removal therapy without the need for medicine and may potentially be used in the treatment of diseases such as haemochromatosis, which require control of iron absorption.
